# Bile salt signaling and bile salt-based therapies in cardiometabolic disease

**DOI:** 10.1042/CS20230934

**Published:** 2024-01-05

**Authors:** Claire C.J. Groenen, Thuc-Anh Nguyen, Coen C. Paulusma, Stan F.J. van de Graaf

**Affiliations:** 1 Tytgat Institute for Liver and Intestinal Research, Amsterdam University Medical Centers, University of Amsterdam, Amsterdam, The Netherlands; 2 Amsterdam Gastroenterology, Endocrinology and Metabolism (AGEM), Amsterdam University Medical Centers, The Netherlands

**Keywords:** ASBT, atherosclerosis, Bile acid, NTCP

## Abstract

Bile salts have an established role in the emulsification and intestinal absorption of dietary lipids, and their homeostasis is tightly controlled by various transporters and regulators in the enterohepatic circulation. Notably, emerging evidence points toward bile salts as major modulators of cardiometabolic disease (CMD), an umbrella disease of disorders affecting the heart and blood vessels that is caused by systemic metabolic diseases such as Type 2 diabetes mellitus (T2DM) and metabolic dysfunction-associated steatotic liver disease (MASLD), the latter encompassing also metabolic dysfunction-associated steatohepatitis (MASH). The underlying mechanisms of protective effects of bile salts are their hormonal properties, enabling them to exert versatile metabolic effects by activating various bile salt-responsive signaling receptors with the nuclear farnesoid X receptor (FXR) and the Takeda G-protein-coupled receptor 5 (TGR5) as most extensively investigated. Activation of FXR and TGR5 is involved in the regulation of glucose, lipid and energy metabolism, and inflammation. Bile salt-based therapies directly targeting FXR and TGR5 signaling have been evaluated for their therapeutic potential in CMD. More recently, therapeutics targeting bile salt transporters thereby modulating bile salt localization, dynamics, and signaling, have been developed and evaluated in CMD. Here, we discuss the current knowledge on the contribution of bile salt signaling in the pathogenesis of CMD and the potential of bile salt-based therapies for the treatment of CMD.

## Introduction

Cardiometabolic disease (CMD) is a group of disorders affecting the heart and blood vessels and is caused by systemic metabolic diseases such as Type 2 diabetes mellitus (T2DM), metabolic dysfunction-associated steatotic liver disease (MASLD), and metabolic dysfunction-associated steatohepatitis (MASH), which share common risk factors such as obesity, dyslipidemia, and high blood pressure [1]. The nomenclatures of MASLD and MASH were recently adopted to overcome the principal limitations of the terms nonalcoholic fatty liver disease (NAFLD) and nonalcoholic steatohepatitis (NASH) [2]. According to the World Health Organization, CMD affects more than one billion people and is the leading cause of death worldwide [1]. Consequently, increasing efforts are made to have a full understanding of the mechanisms behind these diseases which is essential for the development of new therapeutic approaches. Emerging evidence points toward bile salts as major modulators of cardiometabolic health. Therefore, the present review will discuss the current knowledge on the role of bile salts in the pathogenesis of CMD and the potential of bile salt-based therapies to treat CMD.

Bile salts, the primary component of bile, have an established role in the digestion and absorption of dietary lipids and fat-soluble vitamins in the small intestine [3]. Bile salts are amphiphilic molecules that allow strong interaction with hydrophobic phases and form micelles in aqueous environments [3]. This unique characteristic enables the emulsification of dietary lipids and their transport across the mucosa of the small intestine [3]. Importantly, bile salts have been shown to have additional functions beyond their involvement in fat digestion. They have been found to act as hormones, exerting versatile metabolic effects and potentially playing a role in the development of CMD [4]. Bile salts act as metabolic regulators through their interaction with the microbiome and through the activation of bile salt receptors, with the nuclear farnesoid X receptor (FXR) and the Takeda G-protein-coupled receptor 5 (TGR5) as most extensively investigated [4,5].

## Bile salt homeostasis

To explore the role of bile salts in cardiometabolic disease, it is crucial to understand their metabolism, their movement in the enterohepatic circulation, and the regulation of their synthesis by bile salt-responsive receptors FXR and TGR5.

### The metabolism and enterohepatic circulation of bile salts

**Figure 1:**
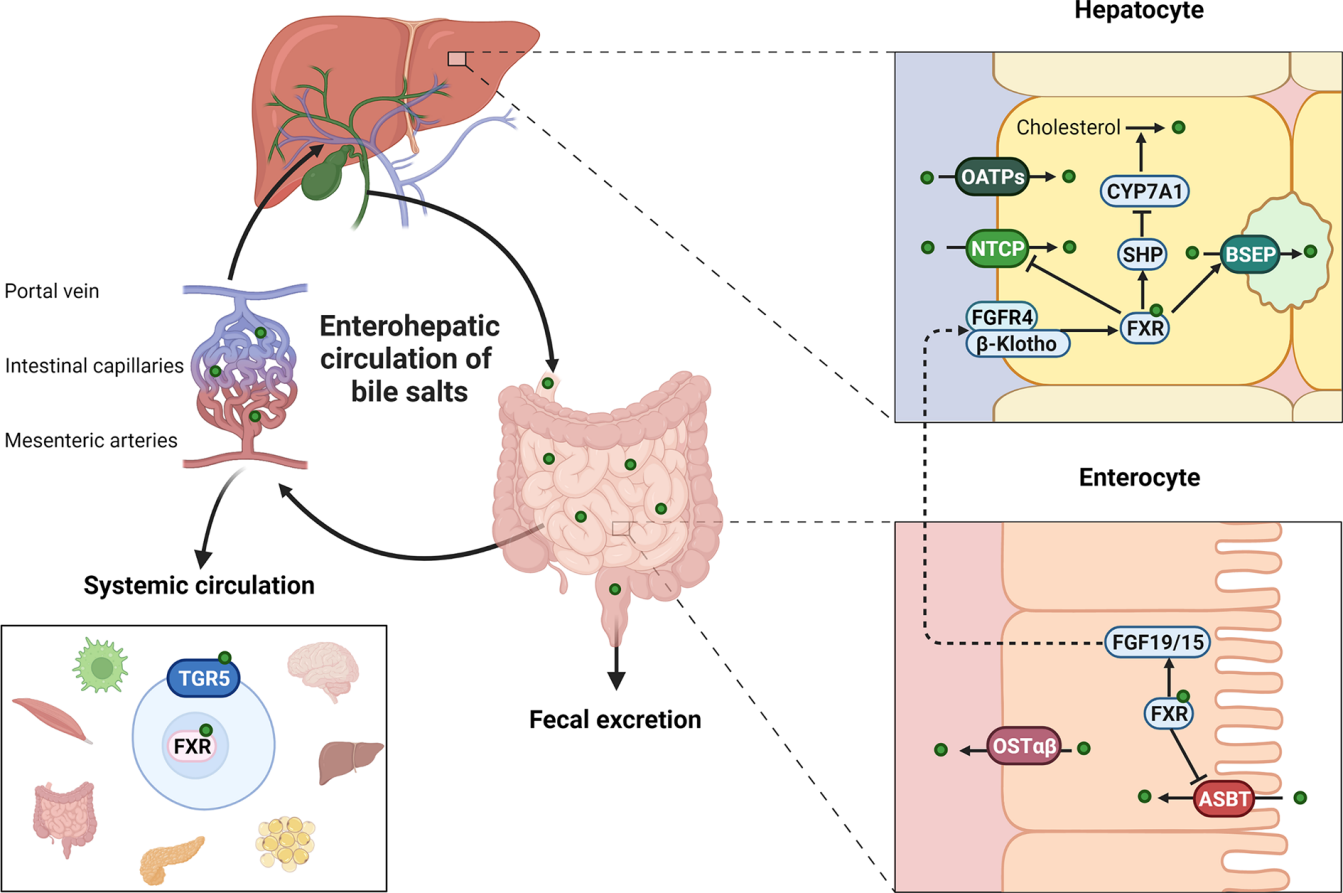
The enterohepatic circulation of bile salts The left side of the figure displays the enterohepatic circulation where bile salts circulate between the liver and intestine via bile and portal blood with a minor fraction spilling over into the systemic circulation. The right side of the figure illustrates bile salt transport within the hepatocyte and enterocyte.

Primary bile salts are biosynthesized in the liver from cholesterol involving two distinct enzymatic pathways including the classical and the alternative pathway, which have been extensively reviewed elsewhere [3]. The classical pathway is initiated by the rate-limiting enzyme cholesterol 7α-hydroxylase (CYP7A1) and accounts for the synthesis of approximately 75% (in mice) and 90% (in humans) of the total bile salt pool, while the alternative pathway is initiated by sterol 27-hydroxylase (CYP27A1) [3,6]. The two major primary bile salts produced in humans include cholic acid (CA) and chenodeoxycholic acid (CDCA), while in mice the two major bile salts include CA and muricholic acid (MCA), the latter being specific for mice and produced via CYP2C70-mediated hydrolysis of CDCA [7]. The synthesis of these primary bile salts is followed by the conjugation with either glycine or taurine [3]. In humans, the ratio of glycine-to-taurine conjugation is 3-to-1, while in rodents more than 95% of the bile salts are taurine conjugated [7]. After synthesis is completed, bile salts are actively secreted from hepatocytes into the canalicular space of the biliary tree by the bile salt export pump (BSEP) and stored in the gallbladder (Figure 1) [4,8]. Upon food intake, the gallbladder contracts, and bile salts are secreted into the intestinal tract where they undergo extensive metabolic conversions by bacterial enzymes such as deconjugation, dehydroxylation, oxidation, and epimerization thereby forming secondary bile salts such as deoxycholic acid (DCA) and lithocholic acid (LCA) in humans, and DCA, Ω-MCA and hycholate in mice [9,10]. When bile salts reach the terminal ileum they are taken up by enterocytes via the apical sodium-dependent bile acid transporter (ASBT) and subsequently excreted from enterocytes by the organic solute transporter complex (SLC51A/B or OSTαβ) into the portal vein to eventually reach the liver where the Na^+^-taurocholate cotransporting polypeptide (NTCP) and the organic anion-transporting polypeptide 1 (OATP1) mediate hepatic uptake [4,8]. A minor fraction of bile salts spill over to the systemic circulation allowing signaling in peripheral organs such as the pancreas, adipose tissue, and the brain [8]. Bile salts cycle 4–12 times a day between the liver and the intestine, in a circulation system referred to as the enterohepatic circulation [8,11] (Figure 1). The daily loss of bile salts is minimized as they remain sequestered in the enterohepatic circulation with a loss of approximately 5% via feces per cycle which is rapidly restored through de novo synthesis thereby maintaining a constant bile salt pool size [3].

### FXR and TGR5 bile salt receptors and bile salt homeostasis

**Figure 2:**
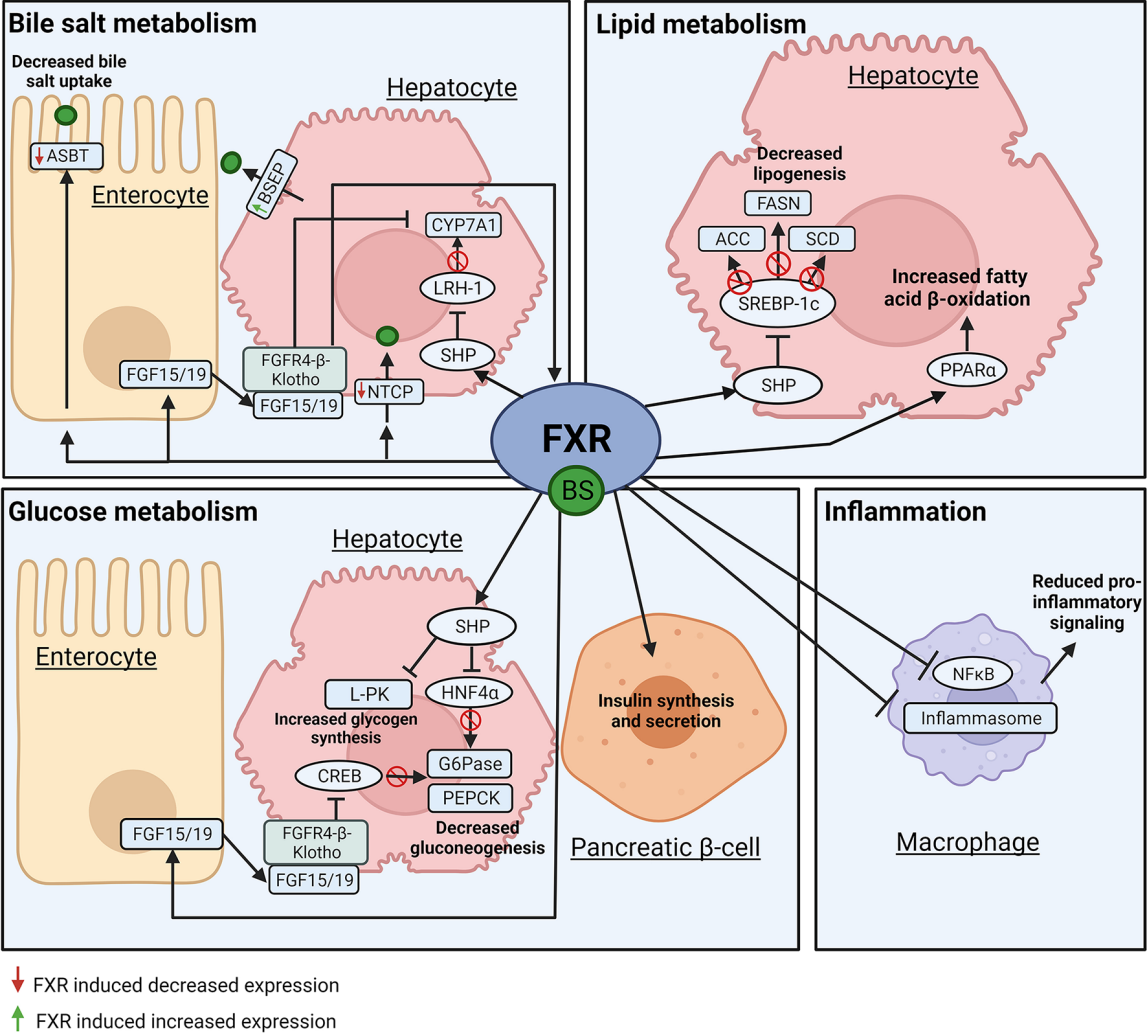
FXR mediated bile salt signaling in inflammation and bile salt, glucose, and lipid metabolism Molecular mechanisms by which the nuclear receptor FXR mediates inflammation and bile salt, glucose and lipid metabolism in enterocytes, hepatocytes, pancreatic β-cells, and macrophages. Transcription factors are oval shaped, while their target genes are rectangular.

The essential role of bile salts in metabolic regulation calls for a tight regulation of their synthesis which is mediated by bile salt-responsive receptors. FXR was first identified in 1995 as an “orphan” nuclear receptor, which is a class of ligand-activated transcription factors regulating gene transcription [12,13]. In 1999, bile salts were shown to bind to FXR and activate FXR-target genes, making FXR the first discovered receptor to be activated by endogenous bile salts [14–16]. CDCA appears to be the most potent agonist to FXR, followed by LCA and DCA, and subsequently CA [17]. Hydrophilic bile salts, such as ursodeoxycholic acid (UDCA) and muricholic acid (MCA), have been shown to antagonize FXR [17]. FXR is highly expressed in the liver, the intestines, the kidneys, and the adrenal gland, and to a lesser extent in other tissues such as white adipose tissue and the heart [18]. FXR activation plays a crucial role in regulating bile salt homeostasis as bile salts exert a negative feedback regulation on their synthesis by activation of FXR (Figure 2). In the liver, FXR activation induces transcription of the nuclear receptor small heterodimer partner (SHP), which suppresses the activity of liver receptor homolog 1 (LRH-1), a transcription factor positively regulating CYP7A1, consequently inhibiting bile salt synthesis [4]. In addition, hepatic FXR activation also limits bile salt accumulation in hepatocytes by inhibiting expression of the bile salt membrane transporter NTCP, and concurrently inducing hepatic bile salt efflux by upregulating expression of the bile salt export pump BSEP [8]. In the intestine, FXR reduces bile salt uptake by reducing expression of ASBT [8]. Intestinal FXR also positively regulates Fibroblast Growth Factor 19 (*FGF19*, *Fgf15* in mice), which is a hormone secreted into the portal circulation, binding to the Fibroblast Growth Factor Receptor 4 (FGFR4)/ β-Klotho complex, present on the plasma membrane of hepatocytes [19]. Activation of this complex results in repression of *CYP7A1* gene expression [19]. Apart from modulating bile salt synthesis and transport, FXR also plays a role in decreasing bile salt toxicity by promoting bile salt biotransformations such as sulfation [20].

**Figure 3:**
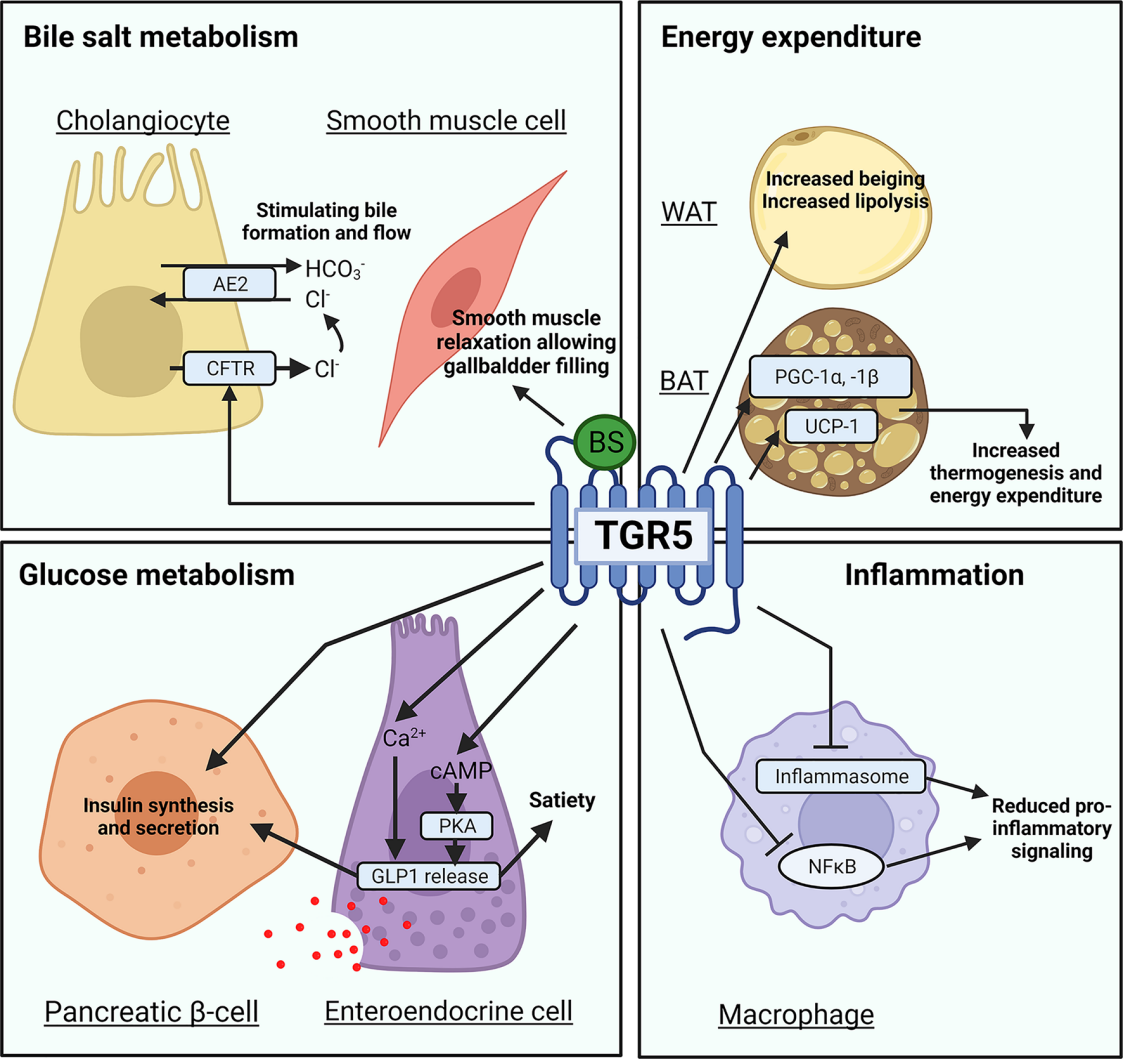
TGR5 mediated bile salt signaling in bile acid and glucose metabolism, energy expenditure, and inflammation Mechanisms by which the GPCR TGR5 mediates bile acid and glucose metabolism, energy expenditure, and inflammation in cholangiocytes, smooth muscle cells, white adipose tissue (WAT) and brown adipose tissue (BAT), enterocytes, pancreatic β-cells, enteroendocrine cells, and macrophages upon bile salt activation. Transcription factors are oval shaped, while target genes are rectangular.

The TGR5 or G-protein-coupled bile acid receptor-1 (GPBAR1) is the most studied bile salt-responsive G-protein coupled receptor (GPCR). The TGR5 receptor was first discovered in 2002 and LCA was shown to be the most potent natural agonist, followed by DCA, CDCA, and CA [21]. Conjugation does not substantially change this order of activation efficacy [22]. TGR5 shows a broad expression in various tissues throughout the body such as the gallbladder, gastrointestinal tract (mainly the ileum and the colon), liver, spleen, kidneys, brown adipose tissue (BAT), skeletal muscle tissue, selected areas of the central nervous system, and monocytes [21–24]. Binding of bile salts to TGR5 results in activation of the adenylyl cyclase-cyclic adenosine monophosphate (cAMP)-protein kinase A (PKA) signaling pathway and subsequent downstream effects which are tissue-dependent [25] (Figure 3). TGR5 signaling plays a role in bile salt homeostasis by regulating bile salt secretion and bile flow in human and mice. TGR5 is extensively expressed in cholangiocytes where its activation promotes chloride (Cl^−^) secretion via regulation of the cystic fibrosis transmembrane conductance regulator (CFTR) (Figure 3) [23]. Cl^−^ secretion provides a gradient that is used by the anion exchanger 2 (AE2) to secrete bicarbonate (HCO_3_^−^) across the apical membrane which protects bile duct cells from bile salt toxicity and enhances bile salt secretion and fluidity (Figure 3) [23]. TGR5 stimulation in mice is also involved in relaxation of smooth muscle cells found in the gallbladder wall thereby allowing the gallbladder to fill with bile (Figure 3) [26]. TGR5-knockout (KO) mice show reduced bile flow and decreased total bile salt pool suggesting a role of TGR5 in bile salt formation, the exact mechanism remains however unknown [27].

## Bile salt alterations in cardiometabolic disease

Monitoring of alterations in bile salt concentrations and pool composition in patients with CMD is essential for understanding how these patients can optimally benefit from therapeutics targeting bile salt signaling. In physiological conditions, fasting serum bile salt concentrations are typically below 5 μM, and may rise to 10–15 μM post-prandially with peak levels 1-2 hours after meal intake [28]. Various studies found an association between CMD and altered plasma bile salt concentrations and pool composition [28]. For instance, in obese patients, fasting bile salt levels tend to be elevated, while postprandial plasma bile salt levels are suppressed [29,30]. In particular, fasting serum concentrations of CDCA and CA and conjugated bile salt levels were found to be elevated in obese patients [29,31,32]. One study found that plasma bile salt levels showed a moderate negative correlation (*r* = −0.30, *P*=0.01) with cognitive restraint of eating in female obese patients (*n*=85), and proposed that increased fasting bile salt levels observed in obese patients may represent a compensatory mechanism to prevent further overeating via TGR5-glucagon-like peptide (GLP1) signaling pathway [29]. Reports on the effect of obesity on fasting total bile salt levels have been, however somewhat inconsistent as also unchanged plasma bile salt levels have been reported [32,33]. Bariatric surgery, such as Roux-en-Y gastric bypass (RYGB) and vertical sleeve gastrectomy (VSG), have shown to be the most effective approach for reducing weight in morbid obesity [34]. Many studies show increased serum bile salt levels after bariatric surgery which is associated with improved glucose metabolism a few days postoperatively, followed by weight loss [35,36]. Bile salts are commonly suggested as mediators of these early improvements in glucose handling and weight loss which may involve bile salt-induced FXR and/or TGR5 signaling [36]. The exact mechanism by which RYGB modifies bile salt metabolism remains unknown. Chávez-Talavera et al. suggested bile salt pool changes after RYGB are potentially caused by alterations in hepatic bile salt recapture as expression of NTCP was reduced after RYGB in minipigs [37]. Various studies have evaluated plasma bile salts and their relation to insulin sensitivity [38–40]. Insulin resistance has shown to positively correlate with particular plasma bile salts, namely, primary and 12α-hydroxylated bile salts [38,39]. In addition, patients with T2DM show elevated levels of taurine-conjugated bile salts [40]. These studies demonstrate a link between dysregulated insulin signaling and altered bile salt pool composition which possibly contributes to metabolic disease development. Higher levels of serum fasting and postprandial bile salt levels are also found in patients with MASLD and MASH [41,42]. Comparing MASH patients to control participants matched for body mass index and insulin resistance revealed that alterations in bile salt concentrations were associated with insulin resistance rather than liver inflammation highlighting the interaction between insulin signaling and bile salts [43]. A positive correlation has been observed between bile salt levels and hypertension in diabetic patients [44]. Furthermore, plasma bile salts were shown to be predictive of systolic blood pressure in men, but not in women [45]. Interestingly, men have a higher total bile salt pool than women [45,46]. Bile salts can affect blood pressure by regulating water and electrolyte homeostasis in the kidney and can thereby play a role in hypertension and CMD development [45]. Lower fasting serum bile salt levels were shown to be highly associated with the presence and severity of coronary artery disease [47,48]. Strong correlations between bile salts and CMD are still lacking and findings often vary across studies due to high inter-individual variability and a small sample size of patients [49]. In addition, mechanisms underlying alterations in plasma bile salt concentration and bile salt pool composition in CMD are incompletely understood, but the diet, genetics, and the microbiome, all likely play a role and more research is warranted [49].

## Mechanisms underlying metabolic effects of bile salts

### Bidirectional interaction between the gut microbiota and bile salt metabolism

Bile salts and the gut microbiota have a bidirectional interaction. The gut microbiota play a major role in bile salt metabolism leading to diversification of the bile salt pool, while bile salts in turn help shape the microbiome [5]. As mentioned previously, the gut microbiota first deconjugate the glycine or taurine moiety of bile salts. The microbial enzymes known as bile salt hydrolases (BSH) are key players in the deconjugation of bile salts and are widely expressed by the major microbial phyla found in the human gut [50]. The physiological function of BSH is still debated but is believed to provide a mechanism for the detoxification of bile salts in some bacterial species by diminishing their detergent properties [51]. This deconjugation reaction allows for additional modifications on the bile salt hydroxy groups by downstream microbial enzymatic reactions thereby creating a wide variety of bile salt-derived molecules [5]. Various microbial-produced bile salt species in mice with unique chemical structures are known such as oxo- and allo-intermediates including 3-oxoLCA and isoalloLCA [52]. In addition, stereoisomers with C-3 hydroxylation in β-configuration, known as iso-bile salts, have been identified in human [53]. Interestingly, iso-bile salts can modulate FXR signaling by functioning as FXR ligands ranging from (partial) agonists to ago-allosteric modulators thereby affecting bile salt synthesis, transport, and metabolism [53]. Microbial modifications can alter the reabsorptive capacity of bile salts and can thereby directly promote or prevent excretion of bile salts via the feces thereby affecting the total bile salt pool [11]. Administration of antibiotics in mice was shown to alter the gut microbiota causing a rapid shift in bile salt pool composition, i.e. a reduction in secondary and an increase in primary bile salts, which highlights the pivotal role of the microbiome in regulating bile salt pool composition [54,55]. TGR5 signaling is particularly affected by alterations in the microbiome, as secondary bile salts including LCA and DCA are the most potent activators of TGR5 [21]. In addition to bile salt modifications, the microbiota can also affect the bile salt pool by regulating their synthesis. As mentioned, the bile salt pool is tightly regulated in the intestine by bile salt-mediated activation of FXR and subsequent induction of FGF19 release which inhibits hepatic bile salt synthesis in humans [19]. Bile salts differ in their ability to activate FXR and therefore in their ability to participate in the negative feedback regulation [17]. For instance, the total bile salt pool size is almost doubled in germ-free mice due to an accumulation of tauro-β-muricholate (TβMCA), which has antagonistic effects on FXR thus inhibiting the negative feedback regulation on bile salt synthesis [56].

Bile salts in turn can directly shape the gut microbial community via their direct and indirect antibacterial properties. Bile salts directly induce antibacterial effects by disrupting their membranes, inducing DNA damage, denaturing proteins, and chelating iron and calcium due to their detergent properties [57]. Indirect antibacterial effects are mediated by the activation of FXR which results in the upregulation of genes whose products (e.g. nitric oxide) are involved in mucosal defense [58]. There is evidence indicating certain bacterial species can develop resistance to bile salts by expression of BSH, by alterations in efflux pumps, and by alterations in membrane lipid and protein position [59–61]. Low levels of bile salts in the gut have shown to be associated with bacterial overgrowth thereby increasing bacterial translocation and inflammation, whilst microbial overgrowth could be reverted by bile salt administration in rats, highlighting the importance of bile salts in maintaining gut microbiota homeostasis [62,63].

The above discussion highlights the bidirectional dependence between the gut microbiota and bile salts described as the microbiota-bile salt axis. As a consequence, dysregulation in either of both can play a role in the development of CMD. Nevertheless, clinical studies investigating the interaction between bile salts, gut microbiome, and CMD are scarce. In a single-blinded randomized controlled trial, 20 male obese subjects received either amoxicillin (*n*=10) or vancomycin (*n*=10) antibiotic treatment (500 mg t.i.d.) for 7 days [64]. Vancomycin but not amoxicillin treatment led to an altered gut microbial fecal composition with reduced levels of the *Firmicutes* bacteria and decreased fecal secondary bile salt concentrations with increased postprandial levels of primary bile salt in plasma, this all coincided with decreased insulin sensitivity [64]. This indicates that the *Firmicutes* bacteria in particular seems to play a role in the metabolism of bile salts and glucose in humans [64]. Another study investigated the mechanism of action of the anti-hyperglycaemic drug metformin, which is frequently prescribed for T2DM patients [65]. This double-blinded randomized clinical trial involved 4-month metformin treatment in treatment-naïve T2DM patients (*n*=22) and led to alterations in the composition and function of gut microbiota, and was associated with increased total as well as unconjugated plasma bile salt concentrations [65]. Transfer of the fecal samples after metformin treatment to germ-free mice showed improved glucose tolerance suggesting a potential link between the anti-hyperglycemic effects of metformin and the gut microbiota-bile salt axis [65]. However, the anti-hyperglycemic effects of metformin are not significantly altered in germ-free and anti-biotic treated mice suggesting the role of the gut microbiota-bile salt axis in the anti-hyperglycemic effects of metformin might be modest [66]. There are a limited number of studies investigating the interaction of the microbiota-bile salt axis in MASLD. For instance, feeding mice with the dietary fiber guar gum has shown to alter the gut microbial community with a reduced abundance of *Deferribacteres* and *Firmicutes* and an increased abundance of *Bacteriodetes*, which was associated with enhanced bile salt levels in plasma and the liver and associated with reduced diet-induced obesity and improved glucose tolerance, while enhancing hepatic inflammation and fibrosis [67]. Depletion of the gut bacteria by antibiotic treatment in mice, diminished portal blood secondary bile salt levels and protected against MASLD [67]. In contrast, another study displayed a protective effect of restoring secondary bile salt levels in a MASH mouse model [68], indicating that the potential link between gut microbiota and MASLD through alterations of bile salts requires further investigation. Finally, a link was found between plasma levels of the microbial-derived product trimethylamine-N-oxide (TMAO) and atherosclerosis development in mice [69]; TMAO affected bile salt metabolism and altered the bile salt profile indicating a link between the gut microbiome, bile salt metabolism, and atherosclerosis development [69].

### Metabolic effects of bile salt signaling

CMD is characterized by various metabolic abnormalities including insulin-resistant glucose metabolism, high plasma cholesterol and triglyceride (TG) levels, and inflammation [1]. Bile salts have shown to exert a variety of downstream effects on cholesterol, lipid, and glucose metabolism, energy expenditure, and inflammation via signaling through the bile salt receptors FXR and TGR5 thereby targeting cardiometabolic abnormalities.

#### FXR bile salt signaling

##### Lipid metabolism

Lipid homeostasis, including TG and cholesterol homeostasis, is a key determinant in the development of atherosclerosis, MASLD, and other cardiometabolic manifestations [70]. FXR is an important regulator of lipid metabolism as FXR KO mice showed elevated levels of hepatic cholesterol and TGs as well as increased plasma bile salt, cholesterol, and TG levels [71]. Watanabe et al. demonstrated in KK-Ay mice, a model for obesity and T2D, that treatment with CA lowered plasma TGs, hepatic TG accumulation, and very-low-density lipoprotein (VLDL) secretion from the liver, which was attributed to the induction of the FXR-SHP axis [72]. The bile salt-induced FXR/SHP pathway has shown to lower TG levels in mice via repression of lipogenic sterol regulatory element-binding protein 1 (SREBP-1c) leading to the inhibition genes involved in lipogenesis including fatty acid synthetase (FASN), acetyl-CoA carboxylase (ACC), and stearoyl-CoA desaturase-1 (SCD) (Figure 2) [72]. Interestingly, Clifford et al., showed in a MAFLD mouse model that FXR activation by the agonist GSK 2324 reduced lipogenic gene expression and hepatic TG levels independently of SHP and SREBP-1c. They also observed a specific shift in bile acid compositions leading to decreased intestinal lipid absorption, which could explain the reduction in hepatic TG levels [73]. In addition, bile salt-induced FXR signaling also promoted lipoprotein lipase (LPL)-mediated clearance of serum TGs via inducing LPL coactivators such as apolipoprotein CII (Apo-CII) and Apo-A5 while repressing the LPL inhibitor Apo-CIII in mice [74,75]. Another study showed that FXR can stimulate fatty acid β-oxidation and prevent hepatic TG accumulation by inducing peroxisome proliferator-activated receptor α (PPARα) activity in human hepatoma cells (Figure 2) [76].

In addition to TGs, FXR also mediates different steps of cholesterol metabolism and thereby plays a role in determining atherosclerotic risk [77]. First, FXR regulates cholesterol breakdown. FXR inhibits CYP7A1 thereby reducing bile salt production from cholesterol [4]. However, CYP7A1 KO mice show that inhibition of CYP7A1 does not result in hypercholesterolemia as cholesterol is eliminated by their secretion into bile by the cholesterol transporter ATP-binding cassette transporter G (ABCG5) and G8 (ABCG8) [18,78]. FXR agonism in mice increases expression of *ABCG5* and *ABCG8* thereby increasing cholesterol elimination via bile [18,78]. In addition, decreased bile salt production due to FXR-induced inhibition of CYP7A1 resulted in decreased intestinal cholesterol absorption and increased excretion via feces [79]. FXR also has other mechanisms for modulating cholesterol levels in plasma and peripheral tissues. Lipoproteins are a transportation form of cholesterol and TGs. FXR activation in human hepatocytes and HepG2 cells resulted in up-regulation of LDL receptor activity through suppressing its negative regulator proprotein convertase subtilisin/kexin type 9 (PCSK9) [80]. FXR agonism also increases reverse cholesterol transport (RCT) in mice, a process that correlates with decreased atherosclerotic lesions, via up-regulating scavenger receptor class B type I (SR-BI) [78,81]. RCT reduces atherosclerosis by returning cholesterol from peripheral cells and tissues including macrophages to the liver for excretion [82]. A study also shows that FXR activation in mice can transactivate phospholipid transfer protein (PLTP) gene expression, which is crucial in the transferring of VLDL to high-density lipoprotein (HDL), further inducing reverse cholesterol transport [83]. Indeed, FXR agonism in mice results in anti-atherogenic effects by enhancing reverse cholesterol transport and increasing cholesterol elimination thereby reducing atherosclerotic plaque formation [79,84]. However, in humans, FXR activation is associated with increased cholesterol levels and pro-artherogenic risk [85].

Interestingly, in atherosclerosis-prone ApoE-deficient mice, FXR inactivation was shown to reduce atherosclerosis development, despite an increase in serum cholesterol and TGs [86]. Here, FXR inhibition attenuated atherosclerosis through reducing expression of CD36, the main transporter in macrophages for the uptake of oxidized LDL, consequently reducing cholesterol accumulation in macrophages and foam cell formation [86]. Another study, however, demonstrated that FXR deletion in ApoE-deficient mice on a high-fat, high-cholesterol diet (HFHCD) increased atherosclerotic lesions together with increased plasma lipids and inflammation [87]. Both foam-cell formation and hypercholesterolemia play a role in atherosclerosis development, and it is likely that the role of FXR in the pathogenesis of this condition depends on the lipid profile of the mice, their diets, and other factors.

##### Glucose metabolism

Evidence suggests an important regulatory role of bile salt mediated-FXR signaling in glucose metabolism as FXR KO mice show elevated levels of plasma glucose and are insulin resistant [88]. Bile salt-mediated activation of FXR has shown to decrease hepatic gluconeogenesis and glycolysis while promoting glycogen synthesis in mice [88]. FXR activation in mouse hepatocytes inhibits the expression of the gluconeogenic enzymes phosphoenolpyruvate carboxykinase (PEPCK) and glucose-6-phosphatase (G6Pase), partly due to repression of the nuclear receptors forkhead box transcription factor 1 (FOXO1) and hepatocyte nuclear factor 4α (HNF4α) (Figure 2) [88,89]. In addition, the intestinal FXR/FGF15/19/FGFR4 pathway also contributes to decreased gluconeogenesis in mice by counteracting cAMP response element-binding protein (CREB) which is a critical regulator of gluconeogenesis (Figure 2) [90]. In addition to gluconeogenesis, FXR was also shown to repress the expression of several glycolytic genes in the liver such as the liver-type pyruvate kinase gene (L-PK), thereby promoting the shift of glucose metabolites from glycolysis towards glycogen synthesis in human hepatocytes (Figure 2) [91]. It has also been reported that FXR is expressed in mouse pancreatic β-cells and directly modulates glucose homeostasis in the pancreas by regulating synthesis, secretion, and insulin sensitivity (Figure 2) [92]. FXR agonism has shown to improve hyperglycemia and insulin resistance in *ob*/*ob* and *db*/*db* mice [89,93]. VSG surgery is unable to decrease body weight and improve glucose tolerance in diet-induced obese FXR KO mice, suggesting an important role of FXR in the beneficial effects of VSG on glucose tolerance and weight loss [94]. In contrast with the above, some studies demonstrated that FXR activation has a negative impact on glucose homeostasis [95,96]. In obese *ob/ob* mice, FXR deficiency was protective against weight gain and peripheral insulin resistance, as shown by improved glucose clearance and adipose tissue insulin sensitivity [95]. This improvement was only shown in whole-body FXR KO but not in hepatic FXR KO mice, potentially emphasizing the significance of FXR deficiency in non-hepatic tissues [95]. The positive effects of intestinal FXR inhibition are not limited to obese animal models. In a recent study, FXR agonist and antagonist were given separately to non-obese diabetic rats after ileotransposition surgery, which involves relocating the distal part of the small intestine between the stomach and the proximal part of the small intestine as a treatment for overweight diabetic patients [96]. The FXR antagonist glycine-β-MCA was found to improve insulin resistance and obesity in rats, while GW4064 FXR agonist resulted in increased plasma glucose levels [96]. It should be noted that effectiveness of glycine-β-MCA on inhibiting intestinal FXR was not assessed in this study, nor potential effects in induced TGR5 signaling. Overall, contradictory studies indicate that the regulatory functions of FXR on glucose homeostasis are complex and possibly involve numerous factors.

##### Inflammation

FXR has been shown to have anti-inflammatory effects as FXR KO mice display strong hepatic inflammation after treatment with lipopolysaccharide indicated by the increase in hepatic necrosis and cytokine signaling of inducible nitric oxide synthase (iNOS), cyclooxygenase-2 (COX-2), and interferon-γ (IFN-γ) [97]. FXR agonism is associated with decreased monocyte chemoattractant protein-1 (MCP-1/CCL2) expression and decreased inflammatory cell infiltration in the liver in a methionine/choline-deficient diet-induced MASH mouse model [98]. FXR's anti-inflammatory properties have been mainly attributed to its transrepressive effects in hepatocytes and macrophages on nuclear factor κB (NFκB), thereby reducing pro-inflammatory cytokines such as tumor necrosis factor α (TNFα), interleukin (IL)-1β, IL6, and iNOS (Figure 2) [58,97]. In addition, FXR has also found the be a negative regulator of the inflammasome by direct interaction with nucleotide-binding domain, leucine-rich–containing family, pyrin domain–containing-3 (NLRP3) and caspase 1 in macrophages (Figure 2) [99].

#### TGR5 bile salt signaling

##### Energy expenditure and body weight

TGR5 has been confirmed as a regulator of energy expenditure as TGR5 KO mice showed faster weight gain and higher fat content compared with wild-type mice [27]. Administration of CA to mice on a high-fat diet (HFD) or CDCA to humans increased energy expenditure in BAT [24,100]. In mice this diet prevented obesity and insulin resistance [24]. TGR5-induced activation of energy expenditure in mice is mediated by up-regulation of uncoupling protein 1 (UCP-1) leading to decreased ATP production as it diverts electron gradients toward thermogenesis thereby increasing energy expenditure in mice (Figure 3) [24]. In line with this, in vitro treatment of human skeletal myocytes and murine brown adipocytes with bile salts increased oxygen consumption [24]. TGR5 activation induces transcription of type 2 iodothyronine deiodinase gene (Dio2) encoding for the enzyme deiodinase type 2 (D2) which potentially plays a role in the observed upregulation of UCP-1 upon TGR5 activation in mice [24]. Other genes involved in energy expenditure including peroxisome proliferator-activated receptor γ coactivator (PGC)-1α and -1β were induced by TGR5 activation in mice (Figure 3) [24]. TGR5 has also shown to play a role in beiging of white adipose tissue (WAT) indicated by enhanced mitochondrial biogenesis and function and increased lipolysis and fatty acid oxidation in thermoneutral housed mice, while this effect was absent in TGR5 KO mice (Figure 3) [101]. A neural role for TGR5 signaling on weight loss has been suggested as TGR5 activation in the brain reduced food intake and body weight gain in mice [102]. In addition, TGR5 signaling induces peptide tyrosine tyrosine (PYY) secretion *in vitro* by intestinal enteroendocrine cells which functions as a centrally active anorectic hormone thereby decreasing food intake and affecting body weight [103].

##### Glucose metabolism

TGR5 is a pivotal regulator of glucose homeostasis by stimulating the secretion of GLP1 by intestinal enteroendocrine cells (Figure 3) [104]. GLP1 is an insulinotropic gastrointestinal hormone that belongs to the family of incretins and their main physiological role is to amplify glucose-dependent insulin secretion from pancreatic β cells after food ingestion [25]. In addition, GLP1 also plays a role in glucose metabolism and energy balance by promoting gastric emptying and acid secretion, delaying intestinal transit, and reducing food intake by enhancing satiety [105,106]. Bile salts have shown to directly induce GLP1 secretion through TGR5 in a murine enteroendocrine cell line [104]. Administration of a TGR5 agonist in TGR5-overexpressing mice on a HFD, improved glucose tolerance, insulin sensitivity, and GLP1 secretion while this effect was absent in TGR5 KO mice [107]. Administration of various TGR5 agonists in mice increases GLP1 secretion and glucose tolerance [108–110]. In contrast, another study found that male TGR5 KO mice showed improved insulin sensitivity on a chow diet, but impaired insulin sensitivity when fed a HFD. Female TGR5 KO mice showed improved insulin sensitivity both on chow and HFD diet, suggesting a gender-dependent regulation of TGR5 function [111]. TGR5 has also shown to be expressed in pancreatic α and β cells [112,113]. TGR5 activation in pancreatic α cells induces pro-convertase-1 thereby promoting GLP1 secretion [112]. TGR5 activation in pancreatic β cells promotes insulin secretion (Figure 3) [113].

##### Inflammation

The first evidence for a role of TGR5 in inflammatory signaling was shown by the inhibition of TNFα secretion in a human monocyte cell line (THP-1) overexpressing TGR5 upon bile salt treatment, which correlated with increased cAMP levels [22]. In line with this, bile salt-dependent TGR5 activation in macrophages, monocytes, and liver resident macrophages known as Kupffer cells, inhibited inflammatory cytokine release, including IL-6, IL-1α, IL-1β, and TNFα, by inhibition of NF-κB after stimulation with lipopolysaccharide (Figure 3) [114,115]. Another study showed that bile salts inhibit NLRP3 inflammasome activation *in vitro* in LPS-primed BMDMs from mice via activation of the TGR5-cAMP-PKA axis, thereby reducing inflammatory signaling (Figure 3) [116]. Primary macrophages obtained from TGR5 KO mice, which were deficient in cAMP production upon treatment with TGR5 agonists, showed an increased production of cytokines further establishing the role of TGR5 in regulating inflammatory responses [117]. In addition to immune cells, TGR5 also functions in mouse endothelial cells that play a role in leucocyte trafficking and inflammation [118]. TGR5 activation induced nitric oxide (NO) production via the phosphorylation of endothelial nitric oxide (eNOS), which protects against oxidative stress and reduces the adhesion of monocytes to vascular endothelial cells *in vitro* [118].

#### Other bile salt signaling mechanisms

Besides TGR5 and FXR-dependent bile salt signaling, various other bile salt-responsive receptors have been identified including the nuclear receptors pregnane X receptor (PXR), vitamin D receptor (VDR), and the constitutive androstane receptor (CAR), as well as muscarinic receptors and the GPCR sphingosine 1-phosphate receptor 2 (S1PR2) [119]. While some of these receptors have shown to have some effects on bile salt metabolism, there is limited data evaluating the biological effects of targeting these receptors in both experimental and clinical settings [119].

Beyond bile salt receptor signaling, a side chain-shortened homologue of UDCA known as nor-UDCA has shown to exert protective effects against primary sclerosing cholangitis and MASLD development independent of bile salt receptors and is currently undergoing clinical evaluation [120]. Nor-UDCA is partly resistant to conjugation with taurine or glycine which enables reabsorption from the bile into cholangiocytes and resecretion by hepatocytes, a process termed cholehepatic shunting [121]. Cholehepatic shunting stimulates HCO_3_^−^‐rich hypercholeresis from cholangiocytes and has shown to have anti-cholestatic, anti-inflammatory, and anti-fibrotic properties [121]. Nor-UDCA has been evaluated in a mouse model of MASLD/MASH and showed direct hepatoprotective, anti-inflammatory, and anti-fibrotic effects [122]. Nor-UDCA combination therapy with drugs targeting FXR/TGR5 bile salt signaling is of interest, as nor-UDCA does not act via FXR or TGR5 activation.

Lastly, various nuclear transcription factors have been identified to regulate bile salt metabolism thereby affecting bile salt signaling [123,124]. Hepatocyte-specific overexpression of activating transcription factor 3 (ATF3) in western-diet-fed LDLR- or ApoE KO mice increased expression of genes involved in cholesterol and bile salt metabolism including SR-BI, LDL receptor (LDLR), ApoE, and Cyp7a1 [123]. In addition, hepatocyte-specific overexpression of ATF3 led to altered bile salt composition which was associated with decreased cholesterol absorption and increased RCT thereby decreasing atherosclerosis development [123]. Another possible regulator of bile salt metabolism is the nuclear receptor corepressor 1 (NCOR1) [124]. In atherosclerosis-prone LDLR-deficient mice on a high-cholesterol diet, hepatic deletion of NCOR1 reduces atherosclerotic lesions and reduces plasma and liver cholesterol contents [124]. Here, NCOR1 deletion attenuating atherosclerosis was attributed to upregulation of Cyp27a1 and Cyp3a11, resulting in altered bile compositions and increased fecal excretion of cholesterol [124]. In contrast, NCOR1 deficiency in macrophages was shown to increase CD36 expression leading to increased foam cell formation [125].

## Therapeutic application of bile salt signaling

Bile salts are potent signaling molecules which play a major role in regulation of glucose, lipid, and energy homeostasis, and inflammation mainly by activation of FXR and TGR5. Various agonists directly activating FXR and TGR5 have been developed and evaluated for their therapeutic potential in CMD. More recently, therapeutics are being developed which inhibit OSTαβ, ASBT, and NTCP bile salt transporters thereby affecting bile salt dynamics and indirectly bile salt signaling [126]. The following subparagraphs will summarize pre-clinical and clinical data of FXR and TGR5 agonists and bile salt transporter inhibitors.

### FXR agonists

Targeting of FXR has been evaluated as a pharmacological strategy for the treatment of CMD due to its downstream effects on lipid and glucose metabolism, and inflammation. FXR agonists can be subdivided into steroidal and non-steroidal agonists [85]. Obeticholic acid (OCA), also known as 6-ethyl-CDCA or INT747, is a steroidal semi-synthetic derivative of CDCA and has shown to be a potent and selective FXR agonist [127]. OCA has been investigated for safety and efficacy in several clinical trials and has been approved by the United States Food and Drug Administration (FDA) for treating primary biliary cholangitis when inadequate response to UDCA is observed [128]. OCA treatment for 72 weeks was able to improve liver histology but also induced hepatic insulin resistance and increased total serum cholesterol and LDL cholesterol levels in MASH patients [129]. OCA is currently evaluated in a global 7-year clinical trial for its effects in MASH patients (NCT02548351) [129], but the Gastrointestinal Drugs Advisory Committee of the FDA has recently expressed concerns with this particular application due to potentially detrimental effects of OCA regarding dyslipidemia, pruritus and risk of cholelithiasis (fda.gov). Various non-steroidal FXR agonists have been developed with the aim to avoid OCA-induced pruritus [85]. GW4064 has shown to be a selective nonsteroidal FXR agonist which improved insulin sensitivity and attenuated hepatic steatosis in different mouse models of obesity and diabetes [88,89,93]. However, GW4064 has poor bioavailability and can potentially induce hepatotoxicity which has halted its evaluation in clinical trials [130,131]. A GW4064 derivative known as Cilofexor was developed with improved pharmacokinetic properties [128]. Cilofexor has been evaluated in a phase II trial with MASH patients and was shown to be safe and significantly improved hepatic steatosis [132]. However, Cilofexor treatment showed only modest beneficial effects on liver biochemistry compared with OCA treatment, indicating potentially limited efficacy [132]. Although non-steroidal FXR agonists may be less strongly associated with pruritus, both non-steroidal and steroidal FXR agonists are associated with decreased HDL and decreased low-density lipoprotein (LDL) cholesterol, which is of concern as patients with metabolic liver disease often show increased cardiovascular risk [85]. Side effects associated with FXR agonists have stimulated the development of intestinal-restricted agonists [128]. It is hypothesized that intestinal-restricted FXR agonists might achieve similar efficacy to systemic FXR agonists while preventing some of the side effects. A nonsteroidal intestinal-restricted FXR ligand known as Fexaramine has shown to ameliorate liver injury and hepatic steatosis in mice on an HFD and shows potential for the treatment of CMD [133,134]. Fexaramine analogs are currently being developed and evaluated for increased efficacy in MASH models [135], but no clinical trials are yet announced.

### TGR5 agonists

The downstream effects on energy expenditure, glucose metabolism, and inflammation associated with TGR5 activation increased interest to explore the therapeutic potential of TGR5 agonists for CMD. Various natural, semi-synthetic, and synthetic TGR5 agonists have been evaluated for their therapeutic application in CMD and have shown promising results. Remarkably, clinical application of TGR5 agonists has been limited, likely due to some unwanted side effects including inhibition of gallbladder emptying, possibly contributing to formation of gallstones, and change in heart rate and blood pressure [26,136].

#### Natural TGR5 agonists

UDCA has shown to be a safe and inexpensive natural bile salt with weak agonistic effects for TGR5 and has antagonist effects towards FXR [137]. UDCA treatment in mice exposed to an HFHCD combined with fructose prevented body weight gain and insulin resistance via TGR5 activation [137]. Clinical trials with UDCA caused a significant reduction in fasting plasma glucose and insulin concentrations thereby improving glucose homeostasis in patients with T2DM and MASLD/MASH, however, UDCA failed to improve liver histology of MASH patients [138–140]. UDCA has immunomodulatory properties as it attenuated liver inflammation and fibrosis in MASH mice [141]. The immunomodulatory properties of UDCA have also shown to be beneficial for the treatment of atherosclerosis. UDCA treatment exerted anti-atherogenic effects by inhibiting pro-inflammatory cytokine production and foam cell formation in macrophages and preventing endothelial dysfunction by blocking endoplasmic reticulum stress and reactive oxygen species production in vitro [142]. In addition, UDCA treatment reduced atherosclerotic plaque formation in diabetic mice [142]. Screening of a library of plant extracts for agonistic effects for TGR5 led to the discovery of oleanolic acid (OA) which can be found in olive oil and many other plants [108]. OA acts as a TGR5 agonist inducing anti-diabetic effects as it lowers serum glucose and insulin levels, enhances glucose tolerance and slows down weight gain in mice on an HFD [108]. OA has also anti-inflammatory effects by suppressing TLR-9, IL-18, and NF- κB signaling pathways in diabetic and obese rats [143,144]. In addition, OA had anti-hyperlipidemic and anti-atherosclerotic effects in both rabbit and mouse animal models of atherosclerosis [145]. It is however unclear whether this anti-atherosclerotic effect is mediated via TGR5 as OA also stimulates other receptors [4].

#### Semi-synthetic and synthetic TGR5 agonists

Low affinity and specificity of natural TGR5 agonists stimulated the design of synthetic TGR5 agonists of which only a limited number reached preclinical testing for CMD treatment thus far. One of the most studied semi-synthetic TGR5 agonists is INT777 which is a derivative of CA. INT777 increased energy expenditure, stimulated GLP-1 secretion and improved insulin sensitivity in mice [107,146]. In addition, INT777 inhibited the inflammasome and macrophage inflammatory signaling in mice [147,148]. INT777 was shown to reduce the development of atherosclerosis in LDLR KO mice by reducing macrophage inflammation and lipid loading [117]. However, clinical trials investigating the therapeutic potential of INT777 are still lacking. SB-756050, a potent and specific TGR5 agonist, is currently the only synthetic TGR5 agonist tested in clinical trials [149]. Short-term daily SB-756050 administration has been evaluated in T2DM patients and was shown to be well tolerated. However, effects on glucose or GLP1 secretion were not dose-dependent and highly variable among participants both within dose groups and between doses. In addition, the lowest doses were associated with an unexpected increase in glucose plasma levels after an oral glucose challenge while no effect on glucose levels was found for the higher doses. The authors hypothesized that lower doses, due to a limited distribution of the compound, caused TGR5 activation predominantly in the proximal gut [149].

### Dual TGR5 and FXR agonists

Various dual TGR5 and FXR agonists have been evaluated as both receptors have shown potential as a target for the treatment of cardiometabolic disease. The semisynthetic steroidal compound INT767 has dual agonistic properties for both TGR5 and FXR [128,150]. It has been reported that INT767 treatment induced FXR-dependent lipid uptake in adipocytes and promoted GLP-1 secretion in enteroendocrine cells by inducing TGR5 [150]. In addition, oral administration of INT767 significantly reduced hepatic steatosis, inflammation, and fibrosis in MASH mice [151]. In ApoE and LDLR KO mice, INT767 treatment significantly decreased serum cholesterol levels and expression of inflammatory cytokines in the aorta by inactivating NF-κB, thereby significantly reducing atherosclerotic plaque formation [152]. In line with this, simultaneous inactivation of TGR5 and FXR exacerbates atherosclerosis in LDLR KO mice [153]. Another synthetic dual TGR5 and FXR ligand known as BAR501 is a derivative of UDCA [154]. BAR501 has been shown to improve insulin resistance, liver histology, and vascular damage, and promoted thermogenesis of BAT in a mouse model of MASH [137,155].

### OSTαβ inhibitors

OSTαβ facilitates the efflux of bile salts from the basolateral membrane of ileal enterocytes into the portal vein to eventually return to the liver. OSTαβ inhibitors are suggested as a strategy to indirectly induce intestine-restricted FXR activation for the treatment of CMD [156]. The therapeutic potential of OSTαβ blockage has previously been evaluated in cholestatic liver disease [156,157]. Challenging OSTα KO mice models with bile duct ligation or cholate feeding showed protective effects against cholestatic liver damage which appears to involve increased urinary bile salt excretion and reduced intestinal bile salt uptake [157]. Pharmacological inhibition of OSTαβ in mice induced intestine-specific FXR activation, and can thereby potentially decrease bile salt synthesis and exert hepatoprotective effects [156]. However, inactivation of OSTα in ApoE and LDLR KO mice on a 16-week atherogenic diet could not attenuate atherosclerosis [158]. Furthermore, the effectivity of OSTαβ inhibitors is questioned as FXR activation can induce OSTαβ up-regulation which will counteract the effect of the inhibitory compound [156]. In addition, concerns have been raised about the intrinsic safety of OSTαβ inhibitors as KO of either OSTα subunit or OSTβ subunit in mice results in a severe ileal phenotype due to intracellular bile salt accumulation [159,160]. In addition, the clinical phenotype associated with OSTαβ deficiency is associated with chronic diarrhea and increased risk for cholestasis [161].

### ASBT inhibitors

The intestinal ASBT is predominantly expressed in the apical membrane of ileal enterocytes and promotes the reabsorption of bile salts from the intestine into the enterohepatic circulation [8,162]. ASBT has been proposed as a potential therapeutic target as inhibition of ASBT increases excretion of bile salts thereby increasing *de novo* synthesis of bile salts from cholesterol [163]. Administration of ASBT inhibitors increases (TGR5-mediated) GLP-1 secretion, significantly decreases *HbA1c* and glucose levels in rats, and may offer a new therapeutic strategy for T2DM [164]. In addition, ASBT inhibitors have been shown to protect against the development of MASLD by restoring glucose tolerance and reducing TG levels in the liver in diet-induced MASLD *in vivo* models [165–167], while clinical trials showed no therapeutic impact [168]. As ASBT inhibitors induce *de novo* synthesis of bile salts they act as cholesterol-lowering agents and thereby protect against the development of atherosclerosis in hamsters and monkeys [169–171]. Various ASBT inhibitors such as Volixibat, Odevixibat, Maralixibat, Lopixibat, Elobixibat, Linerixibat, A4250, and GSK2333072 have entered clinical trials of which Maralixibat and Odevixibat have been approved for commercial development in the EU and USA for the treatment of Alagille’s disease and progressive familial intrahepatic cholestasis (PFIC) [172]. Commercial development of other ASBT inhibitors in particular for application in non-cholestatic diseases has been halted as some had limited efficacy and are associated with adverse side effects such as diarrhea, abdominal pain, and nausea [173–175]. More research should be performed on how to overcome side effects while exerting therapeutic effects. Matye et al. found that combination therapy of the ASBT inhibitor GSK2333072 with FGF15 was overall more effective against MASH and fibrosis compared with monotherapy in mice and might potentially reduce side effects [176]: ASBT inhibitors cause increased bile salt concentrations in the colon which stimulate colon motility and secretion of mucus and water resulting in diarrhea. Co-administration of FGF15, which inhibits bile salt synthesis and thus reduces intestinal bile salt content, counteracts the effects of ASBT antagonism which might prevent adverse side effects such as diarrhea [176]. In addition, a recent clinical trial with an FGF19 analog, Aldafermin, showed to be a promising drug for the treatment of bile acid diarrhea in irritable bowel syndrome patients [177]. Further research is needed to confirm whether combination therapy of ASBT inhibitors and FGF19 may lead to reduced adverse events but pre-clinical data seems promising.

### NTCP inhibitors

NTCP is the main hepatic uptake transporter that mediates the uptake of circulating bile salts from the portal blood into the liver [8]. Inhibition of NTCP has been proposed as a potential treatment for CMD as it allows for transiently elevated bile salt levels thereby prolonging the positive metabolic signaling effects of bile salts in peripheral tissues [178]. Myrcludex B is a synthetic peptide shown to effectively inhibit NTPC-mediated bile salt transport and temporarily increase systemic bile salt levels in humans upon subcutaneous injection [179,180]. Myrcludex B was originally developed for the treatment of hepatitis B and delta virus which infect hepatocytes upon specific docking to NTCP [179]. Treatment of Myrcludex B in obese mice temporarily increased plasma total conjugated bile salts levels 3–4 h after Myrcludex B injection while levels were completely normalized 24 h after the injection [178]. The increase in plasma-conjugated bile salt levels was associated with 4-fold increased GLP-1 fasting levels, increased body temperature, and RNA expression of UCP1 in BAT indicating increased thermogenesis [178]. In addition, Myrcludex B treatment in obese mice reduced body weight, fat mass, and liver steatosis [178]. Decreased levels of serum total cholesterol and LDL-cholesterol were found in patients with NTCP deficiency [181]. In line with this, a clinical trial evaluating 12-week Myrcludex B treatment in hypercholesterolemic volunteers led to a non-significant reduction in LDL-cholesterol levels [182]. Even though this effect was not statistically significant, this study was also underpowered, and more research with larger study populations is needed. Although clinical data on Myrcludex B treatment in CMD is limited, NTCP may provide an interesting target and orally available NTCP inhibitors are under development increasing therapeutic options [183,184].

## Conclusion and future perspectives

Bile salt-based treatments have a longstanding history as dried bear bile, which contains UDCA, was utilized in traditional Chinese medicine for anti-inflammatory and hepatoprotective purposes [85]. Evidence from the past decades has further supported the essential role of bile salt signaling in cardiometabolic health and disease. More synthetic chemical alternatives have been developed, which completely prevent the use of animal-derived bile for therapeutic purposes. More recent efforts to develop bile salt-based drug therapies currently involve bile salt receptor ligands and drugs targeting bile salt transporters in the enterohepatic circulation. FXR and TGR5 agonists have great promise but are also associated with unwanted side effects and more research on how to overcome these side effects is warranted. In addition, bile salt transporters appear as interesting targets to increase systemic bile salt levels and prolong signaling effects of bile salts. This however prompts the need to monitor alterations in bile salt concentrations, pool composition, and levels in patients with CMD to understand how these patients can optimally benefit from therapeutics targeting bile salt signaling. Lastly, more investigation is needed to identify the role of the microbiota-bile salt axis in the development of CMD as dysbiosis and subsequent altered bile salt signaling potentially plays a role in the pathogenesis of CMD.

## Data Availability

This is a review, no primary data is included.

## Abbreviations

ABCG: ATP-binding cassette transporter G
ACC: acetyl-CoA carboxylase
AE2: anion exchanger 2
Apo: apolipoprotein
ASBT: apical sodium-dependent bile acid transporter
ATF3: activating transcription factor 3
BAT: brown adipose tissue
BSEP: bile salt export pump
BSH: bile salt hydrolases
CA: cholic acid
cAMP: adenylyl cyclase-cyclic adenosine monophosphate
CAR: constitutive androstane receptor
CDCA: chenodeoxycholic acid
CFTR: cystic fibrosis transmembrane conductance regulator
Cl^−^: chloride
CMD: cardiometabolic disease
COX-2: cyclooxygenase-2
CREB: cAMP response element-binding protein CREB
CYP27A1: sterol 27-hydroxylase
CYP7A1: cholesterol 7α-hydroxylase
D2: deiodinase type 2
DCA: deoxycholic acid
Dio2: Type 2 iodothyronine deiodinase gene
eNOS: endothelial nitric oxide
FASN: fatty acid synthetase
FDA: Food and Drug Administration
FGF: fibroblast Growth Factor
FGFR4: fibroblast Growth Factor Receptor 4
FOXO1: Forkhead box transcription factor 1
FXR: Farnesoid X receptor
G6Pase: glucose-6-phosphatase
GLP1: glucagon-like peptide
GPBAR1: G-protein-coupled bile acid receptor-1
GPCR: G-protein coupled receptor
HCO_3_^−^: bicarbonate
HDL: high-density lipoprotein
HFD: high-fat diet
HFHCD: high-fat, high-cholesterol diet
HNF4α: hepatocyte nuclear factor 4α
IFN-γ: interferon-γ
IL: interleukin
iNOS: nitric oxide synthase
KO: knockout
L-PK: liver-type pyruvate kinase gene
LCA: lithocholic acid
LDL: low-density lipoprotein
LDLR: low-density lipoprotein receptor
LPL: lipoprotein lipase
LRH-1: liver receptor homolog 1
MASH: metabolic dysfunction-associated steatohepatitis
MASLD: metabolic dysfunction-associated steatotic liver disease
MCA: muricholic acid
MCP-1/CCL2: monocyte chemoattractant protein-1
NASH: nonalcoholic fatty liver disease
NCOR1: nuclear receptor corepressor 1
NFκB: nuclear factor kappa B
NLRP3: nucleotide-binding domain, leucine-rich–containing family, pyrin domain–containing-3
NO: nitric oxide
NTCP: Na^+^-taurocholate cotransporting polypeptide
OA: oleanolic acid
OATP1: organic anion-transporting polypeptide 1
OCA: obeticholic acid
PCSK9: proprotein convertase subtilisin/kexin type 9
PEPCK: gluconeogenic enzymes phosphoenolpyruvate carboxykinase
PFIC: progressive familial intrahepatic cholestasis
PGC: peroxisome proliferator-activated receptor γ coactivator
PKA: protein kinase A
PLTP: phospholipid transfer protein
PPARα: peroxisome proliferator-activated receptor α
PXR: pregnane X receptor
PYY: peptide tyrosine tyrosine
RCT: reverse cholesterol transport
RORγt: retinoid-related orphan receptor-γt
RYGB: Roux-en-Y gastric bypass
S1PR2: sphingosine 1-phosphate receptor 2
SCD: stearoyl-CoA desaturase-1
SHP: small heterodimer partner
SLC51A/B or OSTαβ: organic solute transporter complex
SR-BI: scavenger receptor class B type I
SREBP-1c: lipogenic sterol regulatory element-binding protein 1
T2DM: Type 2 diabetes mellitus
TG: triglyceride
TGR5: Takeda G-protein-coupled receptor 5
THP-1: human monocyte cell line
TMAO: trimethylamine-N-oxide
TNFα: tumor necrosis factor α
TβMCA: Tauro-β-muricholate
UCP-1: uncoupling protein 1
UDCA: ursodeoxycholic acid
VDR: vitamin D receptor
VLDL: very-low-density lipoprotein
VSG: vertical sleeve gastrectomy
WAT: white adipose tissue
